# Articular Cartilage Aging-Potential Regenerative Capacities of Cell Manipulation and Stem Cell Therapy

**DOI:** 10.3390/ijms19020623

**Published:** 2018-02-22

**Authors:** Magdalena Krajewska-Włodarczyk, Agnieszka Owczarczyk-Saczonek, Waldemar Placek, Adam Osowski, Joanna Wojtkiewicz

**Affiliations:** 1 Department of Rheumatology, Municipal Hospital in Olsztyn, 10-900 Olsztyn, Poland; 2 Department of Internal Medicine, School of Medicine, Collegium Medicum, University of Warmia and Mazury, 10-900 Olsztyn, Poland; 3 Department of Pathophysiology, School of Medicine, Collegium Medicum, University of Warmia and Mazury, 10-900 Olsztyn, Poland; adam.osowski@uwm.edu.pl (A.O.); joanna.wojtkiewicz@uwm.edu.pl (J.W.); 4 Department of Dermatology, Sexually Transmitted Diseases and Clinical Immunology, School of Medicine, Collegium Medicum, University of Warmia and Mazury, 10-900 Olsztyn, Poland; aganek@wp.pl (A.O.-S); w.placek@wp.pl (W.P.); 5 Laboratory for Regenerative Medicine, School of Medicine, Collegium Medicum, University of Warmia and Mazury, 10-900 Olsztyn, Poland

**Keywords:** aging, articular cartilage, cell manipulation, stem cells

## Abstract

Changes in articular cartilage during the aging process are a stage of natural changes in the human body. Old age is the major risk factor for osteoarthritis but the disease does not have to be an inevitable consequence of aging. Chondrocytes are particularly prone to developing age-related changes. Changes in articular cartilage that take place in the course of aging include the acquisition of the senescence-associated secretory phenotype by chondrocytes, a decrease in the sensitivity of chondrocytes to growth factors, a destructive effect of chronic production of reactive oxygen species and the accumulation of the glycation end products. All of these factors affect the mechanical properties of articular cartilage. A better understanding of the underlying mechanisms in the process of articular cartilage aging may help to create new therapies aimed at slowing or inhibiting age-related modifications of articular cartilage. This paper presents the causes and consequences of cellular aging of chondrocytes and the biological therapeutic outlook for the regeneration of age-related changes of articular cartilage.

## 1. Introduction

The motor system is the basis for the motion of the human body and the processes that take place in it during aging result not only in limiting the self-sufficiency in performing everyday activities, changes in attitude and gait and increased risk of falling but they also affect the function of internal organs and the quality of life [1].

Attempts to elucidate the causes of age-related changes in articular cartilage and the development of osteoarthritis (OA) include several theories, including the wear and tear theory [2]. Causes of OA development are also sought in processes related to cellular aging, such as the acquisition of the so-called senescence-associated secretory phenotype (SASP), which derives from a change of gene expression in old cells [3], the weakening of the chondrocyte response to growth factors, including the insulin-like growth factor-1 (IGF-1) and transforming growth factor-β (TGF-β) [4], mitochondria function disorders and the effect of oxidative stress [5] as well as the accumulation of advanced glycation end products (AGEs) [6]. Therefore, the destruction of articular cartilage which progresses with age is not only a result of mechanical overload caused by obesity, posture, gait disorders or trauma. Depletion of cartilage that penetrates into the sub-cartilage bone is a gate to non-differentiated mesenchymal cells which—depending on numerous local factors—can differentiate towards osteoblasts which produce bone tissue, towards fibroblasts which contribute to the formation of fibrous connective tissue or towards chondroblasts, which can produce fibrous or hyaline-like cartilage [7]. Unfortunately, physiological regenerative capability of articular cartilage is highly limited and, in cases of significant damage, only external therapeutic intervention can improve the local condition.

## 2. Cellular Senescence

Cellular senescence affects all cells. After the number of divisions (ca. 30–60) determined by the replication limit (Hayflick’s limit) in an in vitro culture, cells lose their replication potential and stop dividing while continuing to age but they do not die at once and can remain metabolically active for a long time [8]. The aging of cells and organs is probably caused by the accumulation of old cells, which—because of changed metabolism and secreted proteins—create their own microenvironment, affecting their own activity and adjacent cells. The low-grade inflammation which develops as a result of these processes accompanies a majority of old-age diseases [9]. Cellular senescence not only reflects the aging of the body but it also plays a significant role in tissue regeneration in young individuals and probably reduces the risk of neoplasm formation by inhibiting mitotic divisions of cells with damaged genetic material [10]. Unlike in apoptotic cells, the activity of senescence associated β-galactosidase (SA-β-gal) increases in the aging process [11]. As a result of DNA damage, aging cells acquire a specific secretory phenotype which leads to their elimination by immune system phages, while at the same time contributing to the development of age-related diseases. The essence of SASP lies in secretion to the environment of a range of cytokines (interleukin: IL-1, -6, -7, -13, -15), inflammatory chemokines (CCL2/MCP-1, CCL8/MCP-2, CCL26, CXCL8/IL-8, CXCL12/SDF-1), growth factors (amphiregulins, EGF, hFGF, HGF, heregulins, KGF, NGF, VEGF), metalloproteinases (MMP) -1, -3, -10, -12, -13, -14, other proteases and their modulators (TIMP-2, PAI-1,PAI-2, t-PA, u-PA) [12].

Two types of cellular senescence are distinguished: replicative and accelerated [13]. Replicative senescence is associated with exhaustion of the division limit. This is caused by shortening of telomeres, whose function is to protect the ends of chromosomes from joining and preserving the genome integrity. Human telomeres consist of thousands of repetitions of motifs made up of six pairs of bases TTAGGG and they constitute a specific counter of cell divisions. The telomere structure is supported by shelterins—proteins that ensure maintenance of their specific structure. Telomeres are multiplied with a specific reverse transcriptase (RT)—telomerase in a complex process, coordinated by genomic replication. Human telomerase is made up of two main subunits—the telomerase RNA template (hTERC) and the catalytic enzyme telomerase reverse transcriptase (hTERT), which are responsible for replication of base pairs in a specific sequence and for the length of telomeres, respectively [14]. Activity of telomerases in regular human tissues is not sufficient to keep the telomere length constant, which results in telomeres becoming shorter with each cellular division [15]. When telomeres are shortened down to half of their original length on five chromosomes, phenotypic cell senescence occurs [16]. Stress-induced premature senescence (SIPS), associated with DNA damage, is another type of cellular senescence that takes place regardless of physiological telomere shortening. Accelerated senescence can be triggered by oxidative stress, oncogenes, UV radiation or a chronic inflammation [17]. This process is faster than replicative senescence and it does not result directly from exhausting the division potential. The causes of each of these types of senescence is different but they are associated with activation of the same path of response to DNA damage. Such a signal in replicative senescence is generated by shortened telomeres or ones without shelterins. A similar response in accelerated senescence is triggered by a rupture of a double strand of DNA in telomere sections which are inaccessible to the repair systems due to their specific structure and protective proteins [17]. A response to DNA damage is controlled, inter alia, with protein p53, which is an inhibitor of cyclin-dependent kinases, which is responsible for inhibiting cellular divisions and hypophosphorylated Rb (retinoblastoma protein), which is responsible for recruiting enzymes associated with epigenetic chromatin modification [18].

## 3. Senescence-Related Changes of Articular Cartilage

**Figure 1:**
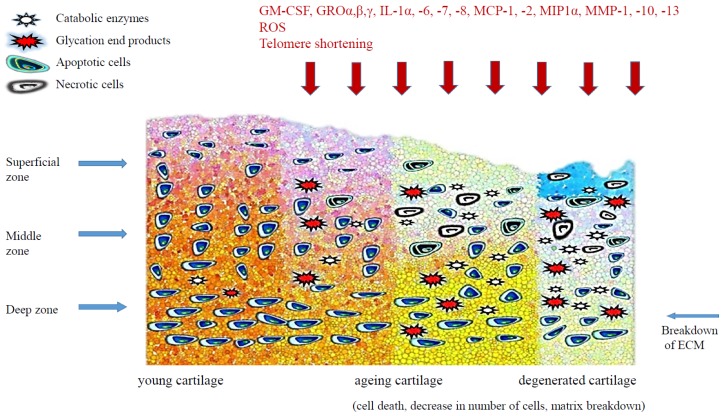
The age-related changes in cartilage.

Homeostasis of cartilage depends on the regularity of function of mature chondrocytes and progenitor cells. With age, the matrix of articular cartilage also undergoes molecular, structural and mechanical changes, there are some changes in the composition and structure of proteoglycans, collagen cross-linking increases and the elongation strength of cartilage decreases. The balance between anabolic activity of chondrocytes and destructive processes is disturbed. Articular cartilage thins slowly as the matrix reduces, cartilage hydration decreases and the chondrocyte count decreases (Figure 1). An age-related decrease in the number of chondrocytes in articular cartilage has been observed in people without clinical symptoms of arthritis, although more pronounced chondrocyte loss is present in patients with OA [19]. The number of chondrocytes in the hip joint cartilage in people aged 30–70 years was reduced by ca. 40% [20]; similar differences have been observed in animal studies [21]. However, in a study of human knee joint cartilage, no significant changes in the number of chondrocytes were observed [22]. The frequency of chondrocyte divisions observed in articular cartilage in adults is low, which—with a very small number of local progenitor cells—may suggest that chondrocytes in elderly people are the same cells as many years earlier but are considerably changed. It also appears that the number of apoptotic cells in human articular cartilage does not increase significantly with progressing age [22].

Senescence of chondrocytes is accompanied by aging-related changes in extracellular cartilage matrix (ECM). The unique properties of extracellular matrix of cartilage have their source in collagenic and non-collagenic glycoproteins, proteoglycans and hyaluronic acid. ECM in articular cartilage plays a crucial role in regulating chondrocyte functions via cell-matrix interaction, organized cytoskeleton and integrin-mediated signalling [23]. A reduction in cartilage volume can be caused by a reduction of water content dependent largely on the content of aggrecan, which is the principal proteoglycan in an articular cartilage matrix. Sulphated, negatively-charged glycosaminoglycans (GAG), which make up aggrecan, are characterised by high hydrophilicity and are responsible for cartilage elasticity. There have been reports describing age-related changes of size, structure and degree of sulphation of aggrecan which resulted in a decrease in hydration and elasticity of cartilage [24].

### 3.1. Telomere Shortening

Like in cells of other tissues, telomeres have been found to be significantly shortened in chondrocytes of articular cartilage which have passed through a larger number of cellular divisions [25]; larger numbers of shortened telomeres have also been reported in chromosomes of chondrocytes in elderly people [25]. Age-dependent shortening of telomeres has been observed recently in a study conducted with people with no symptoms of arthritis and in a group of OA patients [26]. The activity of telomerase is higher in chondrocytes in young individuals, which enables repair procedures in young chondrocytes; this activity is reduced considerably after puberty [27]. Attempts have been made to explain the importance of telomeres shortening for senescence of chondrocytes and their precursors—mesenchymal stem cells (MSCs). The length of telomeres varies depending on the donor’s age. Embryonic or foetal cells have longer telomeres, telomerase activity in them is higher and they senesce later than cells collected from adult individuals [28]. The length of telomeres in chondrocytes has been reported as 9 to 11 kbp in donors over 55 years old and below 12 kbp in donors under 22 years old [3]. Guillot et al. report that telomeres in foetal stem cells were significantly longer (10 to 11 kbp) than in MSCs obtained from the bone marrow of adult individuals (under 7 kbp) [28], whereas Mareschi et al. found the length of telomeres in MSCs of young donors to be approx. 10 kbp [29]. In these in vitro studies, the telomere length in bone marrow MSCs decreased by 1.5–2 kbp per passage. Telomere length in bone marrow MSCs decreased by 17 bp a year, as found in an in vivo study [30]. Interesting findings were presented in a paper by Parsch et al. where telomeres in bone marrow MSC remained shorter than in chondrocytes even after they were chondrogenically differentiated and their shortening was inhibited at a length of approx. 10 kbp [31]. Apart from the length reduction caused by replicative senescence, telomeres shortening is caused by oxidative stress and destruction of DNA strands [32]. Telomeres in chondrocytes and in MSCs have been found to shorten considerably in cell culture subjected both to sublethal oxidative stress and to prolonged low-level stress [33,34]. A relationship has been described between a considerable shortening of telomeres in chondrocytes and intensified senescence of chondrocytes and the severity of OA [35], although no differences have been observed in another study in the length of telomeres in regular chondrocytes and in those collected from OA lesions [36]. The reason for the large differences between these studies may be the use of different telomere length estimation methods.

### 3.2. Oxidative Stress

Chondrocytes and MSCs are known to be exposed naturally to under-physiological oxygen concentrations. Reactive oxygen species (ROS) induce telomere shortening stimulated by DNA damage [37,38]. The amount of ROS in chondrocytes increases with age, excessive mechanical load and activity of inflammatory cytokines [39]. The addition of ROS to a chondrocyte culture resulted in developing aging-related phenotypic traits in chondrocytes [39]. The amount of proteins (which are shelterins) associated with telomerase TRF1, TRF2 (telomeric repeat binding factor 1, 2) increases considerably in chondrocytes under oxidative stress during early cell division. These proteins are responsible for the formation and maintaining the structure of telomeres, protein XRCC5 (X-ray repair complementing defective repair in Chinese hamster cells 5) participating in the repair of two-strand DNA and sirtulin 1 (SIRT1), which suppresses protein p53 and prevents inhibition of cell divisions. Secretion of these shelterins is much weaker in later divisions [37]. This study suggests a protective effect of proteins TRF1, TRF2, XRCC5 and SIRT1 on young chondrocytes against shortening of telomeres associated with damage to DNA strands under oxidative stress, whereas in chondrocytes which divide later, a decrease in the activity of these regulatory proteins results in decreased tolerance to ROS and in the accumulation of damaged DNA, which may induce aging-related processes. ROS appear to induce acceleration of chondrocyte senescence by intensifying expression of p53 and p21 and by activation of p38 MAPK (mitogen-activated protein kinase) and phosphatidylinositol 3-kinase/Akt (PI3K/Akt) signalling pathways [40]. Chondrocytes in areas of cartilage affected by OA have been found to contain an increased amount of nitrotyrosine—an oxidative damage marker—which was proportionate to intensification of histological changes [41]. Oxidative stress affecting chondrocytes participates in inducing apoptosis, decreases the cells sensitivity to growth factors, leads to mitochondria dysfunction, telomere-related genomic instability and loss of cartilage matrix [42,43,44,45]. ROS also contribute to senescence of MSCs, which are precursors of chondrocytes. The presence of antioxidants (such as *N*-acetylcysteine (NAC) and ascorbic acid) in cell cultures, increased the proliferative activity of MSCs [44,46] and the proliferative and differentiating capability of MSCs in low oxygen concentration (3–5%) was higher than in physiological concentrations (20%) [47,48].

With age, excessive formation of ROS takes place and the oxidative-antioxidative equilibrium is disturbed in cartilage matrix. In reaction with the core protein of proteoglycans, reactive oxygen species modify amino acid residues and cause ruptures of the polypeptide chain of the core protein and formation of proteoglycan fragmentation products in the form of glycosaminoglycan chains bound to core protein residues and free glycosaminoglycans [49].

### 3.3. Inflammatory Cytokines

Epidemiological studies indicate a relationship between a low-level systemic inflammation and an increase in a concentration of inflammatory cytokines, including C-reactive protein (CRP), IL-6 and TNF-α and the development of OA [50,51]. Inflammatory cytokines can be generated locally in chondrocytes, synovial membrane cells and infrapatellar fat pad-derived cells [50]. Articular cartilage aging may result from the acquisition of a specific secretory phenotype by chondrocytes, whose characteristic features include an increase in the production and secretion of interleukins, matrix metalloproteinases and growth factors, including epidermal growth factor (EGF) [38,52]. SASP-inducing factors include granulocyte macrophage colony stimulating factor (GM-CSF), growth regulated oncogene-α, -β, -γ (GRO-α, -β, -γ), IL-1α, IL-6, IL-7, IL-8, monocyte chemoatractant protein (MCP)-1, -2, IGF-1, macrophage inflammatory protein-1α (MIP-1α) as well as MMP-1, MMP-10 and MMP-13 [50]. Literature reports have described increased expression of metalloproteinases MMP-1 and MMP-13 in aging cartilage [53] and the accumulation of neoepitopes of collagens formed as a result of denaturation and fragmentation of collagen [54]. Other studies have found the capability for production and secretion of IL-1 [55] and IL-7 [56] by isolated chondrocytes to significantly increase with the donor’s age and an increase in IL-7 secretion to be associated with increased production of MMP-13 [56]. Moreover, the inflammation process induced by the administration of IL-1β was associated with an increase in the expression of p16^INK4a^, resulting in increased production of MMP-1 and MMP-13 [52].

The total amount of all proteoglycans in cartilage decreases with age. Age-related modifications of proteoglycans in cartilage matrix are related to synthesis disorders and enzymatic and non-enzymatic degradation. Degradation of proteoglycan macromolecules which intensifies with age is accompanied by overexpression of MMP, including MMP-1, MMP-8, MMP-13 with a concomitant decrease in their tissue inhibitors (TIMP) [57]. The activity of disintegrin and metalloproteinase with thrombospondin motifs (ADAMTS), including aggrecanases ADAMTS-4 and ADAMTS-5, which participate in the digestion of core proteins of aggrecans, increases with age [58]. Apart from a direct effect degrading the extracellular matrix of cartilage, these enzymes stimulate the secretion of inflammatory cytokines, e.g.,: IL-1, IL-6 and the tumour necrosis factor-α (TNF-α) [38]. IL-1, IL-6 and TNF-α induce chondrocytes to synthesise increased amounts of matrix metalloproteinases, at the same time inhibiting the production of natural inhibitors of these endopeptidases [58]. Additionally, IL-1 and TNF-α stimulate the production of insulin-like growth factor-binding protein-1 (IGFBP-1). IGFBP-1 binds IGF-1, thereby decreasing its binding capability with an appropriate receptor on chondrocytes, which leads to a decreased response of mature chondrocytes to IGF-1 [59]. An effect of IL-1 and TNF-α results in an increase in the activity of inducible nitric oxide synthase (iNOS), and—secondarily—in an increase in secretion to the matrix of catabolic metalloproteinases and prostaglandins [58].

### 3.4. Altered Responsiveness to Growth Factors

With time, the anabolic activity of chondrocytes in cartilage decreases and the chondrocyte metabolic equilibrium shifts towards catabolic mechanisms.

Chondrocyte response to IGF-1 decreases with age [4]. Similar disturbances also occur in isolated chondrocytes from cartilage with symptoms of OA [60]. IGF-1 stimulates the proliferation of cartilage cells, supports the synthesis of cartilage matrix cells and inhibits chondrocytes apoptosis through phosphoinositide 3-kinase (PI3K) and extracellular signal-regulated kinase (ERK) [61]. IGF-1 increases the synthesis of proteoglycans of cartilage matrix under in vitro conditions by activating the kinases PI3K/Akt/mTOR/p70S6 pathway [62]. Through PI3K, IGF-1 stimulates MSCs to chondrogenic differentiation [62].

Expression and amount in cartilage of osteogenic protein OP-1 (BMP-7), which is a member of the bone morphogenetic proteins superfamily, decreases with age. The addition of anabolic protein OP-1 to a culture of chondrocytes collected from mature individuals does not affect the activity of telomerase, whereas an addition of inflammatory cytokine IL-1α inhibits its activity [27].

The concentration of TGF-β2 and TGF-β3 (but not TGF-β1) in cartilage also decreases in an age-related manner, like the number of TGF-β receptors [21]. TGF-β is secreted by cells as a latent form (latent TGF-β, l-TGF-β). An active form is generated after dissociation of the non-covalently bonded latency-associated peptide (LAP). LAP dissociation is effected by the plasminogen/plasmin proteolytic system [63], thrombospondin-1 (TSP-1) [64], metalloproteases [65] as well as by mechanical stress [66]. After TGF-β binds to the II type receptor, an activin receptor-like kinase 5 (ALK5) recruiting complex is formed, which is a TGF-β type I receptor. Such a compound induces phosphorylation of serine and threonine residues of type I receptor by type II receptor. The primed receptor transmits the signal directly to cytoplasm, where R–SMAD proteins are phosphorylated and translocated to the cell nucleus [67]. TGF-β signal transmission can be mediated by an alternative ALK1 type I receptor, resulting in the final cellular differentiation and hypertrophy [68]. Binding of TGF-β to the ALK5 receptor leads to phosphorylation of proteins: SMAD2 and SMAD3, whereas binding of TGF-β to the ALK1 results in phosphorylation of SMAD1, SMAD5 and SMAD8. Activation of SMAD2/3 and SMAD1/5/8 pathways differs by the response. Signal transduction by SMAD2/3 is associated with a protective effect and by SMAD1/5/8—with terminal differentiation and hypertrophy [69]. Activation of signal pathways is necessary for in vitro development of MSCs population. Inhibition of TGF-β pathways in rat and human cultures prevented their differentiation [70]. TGF-β in MSCs cultures can be a factor used in the induction of chondrogenesis [71]; on the other hand, TGF-β can accelerate processes of cellular senescence by increasing the activity of senescence-associated-galactosidase (SA-Gal) and the production of mitochondrial reactive oxygen species (mROS) [72]. Philipot et al. found that—during chondrogenic differentiation of BM-MSCs caused by TGF-β3—expression of p16^INK4a^ accompanying the production of type IIB collagen and MMP13 took place, which was a sign of terminal cell differentiation [52].

### 3.5. Advanced Glycation End-Products

Modifications of proteoglycans of articular cartilage taking place with time are caused by the catabolic effect of enzymes and oxidative stress but also by accumulation of products of late glycation in cartilage (advanced glycation end-products, AGEs). The production of AGEs takes place as a result of spontaneous, non-enzymatic glycation of proteins which, in turn, is a result of reaction of reducing sugars, including sucrose, fructose and ribose with lysine and arginine residues. Due to its relatively slow metabolism, cartilage is particularly predisposed to form AGEs. The half-life of type II collagen, which is the most widespread protein of extracellular cartilage matrix, has been estimated to exceed 100 years [73]. Although products of late glycation decrease the sensitivity of proteoglycans to proteolytic effect of metalloproteinases, the total pool of proteoglycans in cartilage is decreased proportionally to the amount of AGEs [74]. The effect of AGEs on processes that take place in aging cartilage has not been fully elucidated. The appropriate receptors (receptor for advanced glycation end products, RAGE) on the chondrocyte cell membrane are probably responsible for inhibition of synthesis and secretion of proteoglycans to cartilage extracellular matrix and for inducing the synthesis of matrix metalloproteinases (MMP-1, MMP-3, MMP-13) and prostaglandine E2 [57].

### 3.6. Autophagy

Autophagy has gained interest in the past decade due to its role in regulation of the aging process. Autophagy is a naturally occurring catabolic process that removes unnecessary of dysfunctional cellular components in cytoplasm as aggregated proteins and redundant or damaged organelles [75]. Little is known about the role of autophagy in articular cartilage. In articular cartilage, which has a very low rate of cell turnover, autophagy appears to be protective process for maintaining cartilage homeostasis. Autophagy regulates maturation and promotes survival of terminally differentiated chondrocytes under stress and hypoxia conditions [76]. Transiently increased autophagy is a compensatory response to cellular stress. During the early degenerative phase, autophagy is increased in cartilage, with increased accumulation of autophagic proteins, such as ULK-1, LC3 and Beclin-1 messenger RNA in chondrocytes [77]. Reduced expression of ULK1, Beclin-1 and LC3 protein was observed in aging joints in humans and mice [78]. The reduction in these major autophagy regulators was accompanied by increased chondrocyte apoptosis [77,78]. Many studies identified correlations between autophagy and the mTOR signalling pathway. The cartilage-specific deletion of mTOR upregulates autophagy and results in increased autophagy signalling and a significant protection from the articular cartilage degradation, apoptosis and synovial fibrosis [79]. The intra-articular injection of rapamycin- an mTOR inhibitor [80], or intra-articular administration of gelatin hydrogels incorporating rapamycin-micelles [81], inhibited mTOR expression suppressed the development of the articular cartilage degeneration. Additionally, REDD1- an endogenous inhibitor of mTOR that regulates cellular stress responses is highly expressed in normal human articular cartilage and reduced with age [82]. Chondrocytes are adapted to hypoxic conditions. Two main HIF hypoxia-inducible factors isoforms (HIF-1α and HIF-2α) mediate the response of chondrocytes to hypoxia. HIF-1α supports metabolic adaptation to a hypoxic environment and by suppression of mTOR causes increased autophagy [83]. In contrast, HIF-2α has been shown to be a suppressor of autophagy under hypoxic conditions in vitro [84].

## 4. Possible Anti-Aging Strategies

Old age and, obviously, the aging of tissues and organs pose a challenge to contemporary medicine. Neither the treatment of pain as the main symptom of changes related to aging of the motor system, nor burdensome surgeries are fully satisfying to patients; hence, the need for alternative methods for slowing down the aging process and supporting regeneration of articular cartilage.

### 4.1. Cell Manipulation

#### 4.1.1. Telomerase Activators

Several studies have been conducted to assess the effect of an increase in the hTERT activity on the lifespan of MSCs. Increased expression of hTERT by transduction in MSCs resulted in extended in vitro cell replication capability and in maintaining the potential for in vitro adipo-, chondro- and in vivo osteogenic differentiation [85,86]. Neither a tendency for neoplasm formation nor changes in the MSCs caryotype were observed in these studies. Recently, several telomerase-inducing factors have been discovered and described [87,88,89]. Astragaloside (AST) and its active metabolite cysloastragenole (CAG) activated telomerase and slowed down the aging process in human embryonic kidney HEK293 fibroblasts [90]. Cynomorium songaricum polysaccharide increased telomerase activity and led to elongation of telomeres in murine cells [91]. Tichon et al. found chemical telomerase activators: AGS-499 and AGS-500 to induce expression of TERT in MSCs, to increase the length of telomeres and to stimulate cell resistance to apoptosis induced by oxidative stress. No chromosomal aberrations or MSCs differentiation disorders were observed [92]. Telomerase differentiation in vitro was increased by curcuminoid derivatives with the core and at least one *n*-pentylpyridine side chain [93]. An extract of Astragalus membranaceus root (TA-65) used in another study extended the proliferative activity of T cells [94] and it significantly decreased the amount of extremely short telomeres in a study of mice [95].

#### 4.1.2. Antioxidants and Hypoxia

Several antioxidants have been described with a protective effect on chondrocytes and MSC against cellular senescence and oxidative stress-induced apoptosis. NAC inhibited apoptosis in chondrocytes [40] and MSCs [44] subjected to oxidative stress. In the study by Liu et al., stimulation of chondrocytes with IL-1β caused a significant up-regulation of TLR4 and its downstream targets MyD88 and TRAF6 resulting in NF-κB activation associated with the synthesis of IL-1β and TNFα. These IL-1β-induced inflammatory responses were all effectively reversed by resveratrol- a polyphenol of plant origin. Furthermore, activation of NF-κB in chondrocytes treated with TLR4 siRNA was significantly attenuated but not abolished and exposure to resveratrol further reduced NF-κB translocation [96]. In another study, following resveratrol injection, the expression of collagen type II was retained but the expression of inducible nitric oxide synthase and matrix metalloproteinase-13 was reduced in OA cartilage. Moreover, the administration of resveratrol significantly induced the activation of SIRT1 expression in mouse OA cartilage and in IL-1β-treated human chondrocytes [97]. The effectiveness of antioxidants in preventing damage to chondrocytes seems to increase when biomaterial scaffolds are used [98]. A cell-free collagen/resveratrol (Col/Res) complex in the form of hydrogel significantly reduced the activity of IL-1β, MMP-13 and COX-2 in an animal OA model; additionally, it improved the condition of articular cartilage after only 12 weeks [99]. The physiological oxygen concentration was found to weaken the growth of human chondrocytes and MSCs in a culture and an increase in antioxidant production as a response to these oxygen concentrations resulted in cell destruction [100,101]. Hypoxia in culturing regular human chondrocytes enabled maintaining the chondrogenic cell potential [102] but this was not observed in a study with osteoarthritic human chondrocytes [103]. In the study by Choi et al. chondrogenic differentiation of ASCs was found to be increased under hypoxia as evidenced by the greater amount of proteoglycan formation and increased chondrogenic genes expression, ACAN, COL2 and SOX9 [104]. It appears that human MSCs acquire aging-related traits in cultures under hypoxic conditions later than under normoxic ones. Additionally, the frequency of MSC divisions increases at low oxygen concentrations with the intact genotype maintained [105]. An increase in the chondrogenic potential of MSCs caused by hypoxia has been reported [104,106], although—as with chondrocytes—the direction of MSCs differentiation depends on the oxygen concentration but also on other factors, such as the substrate used [107,108].

#### 4.1.3. Mechanical Load

Mechanical load stimulates the metabolic activity of chondrocytes, which is responsible for keeping the metabolic balance of matrix proteins [109] and participates in processes which regulate MSCs differentiation towards chondrogenesis [110]. Dynamic compressive loading is the most common system of mechanical stimulation of MSC-dependent cartilage regeneration used in mechanobiological studies. The process of mechano-transduction is, at least partly, mediated by the TGF-β signalling pathway [111,112,113]. Synthesis of matrix proteins by MSCs stimulated by multiaxial pressure is more effective than with monoaxial stimulation [114]. Multidirectional forces support the production of type II collagen, aggrecan and GAG, with or without the participation of external growth factors, including TGF-β [113]; intensification of the production and activity of endogenous TGF-β caused by mechanical load has been reported [111]. In a human MSCs culture, dynamic compressive loading significantly inhibited the expression of hypertrophy markers, such as type X collagen X, MMP-13 and alkaline phosphatase (ALP) [114]. In another study, mechanical inhibition of chondrocyte hypertrophy was mediated by TGF-β/SMAD and integrin β1/focal adhesion kinase (FAK)/extracellular-signal-regulated kinase (ERK) pathways [115]. These findings suggest that applying a mechanical load to a joint following the implantation of cartilage obtained by bioengineering or applying a load to cellular structures before intra-articular implantation can support cartilage maturation and inhibit its hypertrophy, giving better results than immobilisation of the joint, which can have significant application in clinical practice.

### 4.2. Cell-Based Therapy

Attempts have been made for a long time to surgically stimulate local regeneration of weakened articular cartilage in such procedures as abrasion arthroplasty, drilling and microfracture. Alternatively, there are therapies based on autologous chondrocyte implantation (ACI), autologous matrix-induced chondrogenesis (AMIC) and intra-articular injection of mesenchymal stem cells [116,117,118,119].

#### 4.2.1. Autologous Chondrocyte Implantation

ACI is an example of the application of tissue engineering in the treatment of small- and medium-size cartilage loss. This technique includes a surgical biopsy of cartilage from an affected joint, isolation of chondrocytes with collagenase and creation of a monolayer culture, which requires a series of cellular passages to achieve sufficient propagation of chondrocytes. At the next stage, cells acquired in vitro are implanted at the site of a loss and immobilised with a periosteal patch, which has a protective role but which also has chondrogenic capability, or they are protected with a collagenic layer [120]. The results of the ACI procedure observed in population studies have been encouraging—pain within the joint decreased and the formation of high-strength tissue was observed [121]. However, there are some limitations to the application of ACI. However, it seems that the proliferative capability and the chondrocytes’ ability to propagate in a one-layer culture decrease with the age of a donor [122]. The study by Barbero et al. did not show any difference in the proliferative activity of chondrocytes collected from donors under 40 years of age, whereas proliferation of chondrocytes from donors over 40 years of age slowed down considerably [123]. A decrease in the proliferative capability of chondrocytes collected from older donors can obviously limit the use of ACI in treating age-related changes. Changes in the phenotype are also an issue in monolayer chondrocyte cultures. The culture conditions do not reflect fully the in vivo conditions. When cultured in vitro, cells can change shape from polygonal or round to flat after as few as four passages; at the time, they start synthesising type X collagen, which is a sign of a loss of the chondrocytal phenotype. With a growing number of passages, aging and dedifferentiation of chondrocytes in a monolayer culture is taking place, which is associated with a decrease in the production of type II collagen, proteoglycans and glycoproteins [40]. Transplantation of old and dedifferentiated cells results in an unwanted growth of fibrous cartilage at the site under treatment [124]. Apart from a conventional monolayer chondrocyte culture, there are methods currently in use using growth factors [62], pellet culture [125] and bioreactors [126]. De-differentiation can be inhibited and chondrogenicity can be regained successfully by functionalization of the dynamic culture surfaces with ECM [127], culture on a continuously expanding surface [128], surface coating with Col I [129] and by applying a mechanical load [130]. Although the methods are effective in maintaining the chondrocytal phenotype, they may not be able to achieve sufficient cell expansion [131]. Much attention has been recently devoted to supplying genes associated with cellular growth factors, including TGF-β1, BMP, IGF-1, fibroblast growth factor (FGF) and transcription factors, i.e., SRY (sex-determining region Y)-box 9 (SOX9), RUNX2 and SMAD; microRNAs and Col II promoter-binding proteins [132].

#### 4.2.2. Mesenchymal Stem Cells

**Figure 2:**
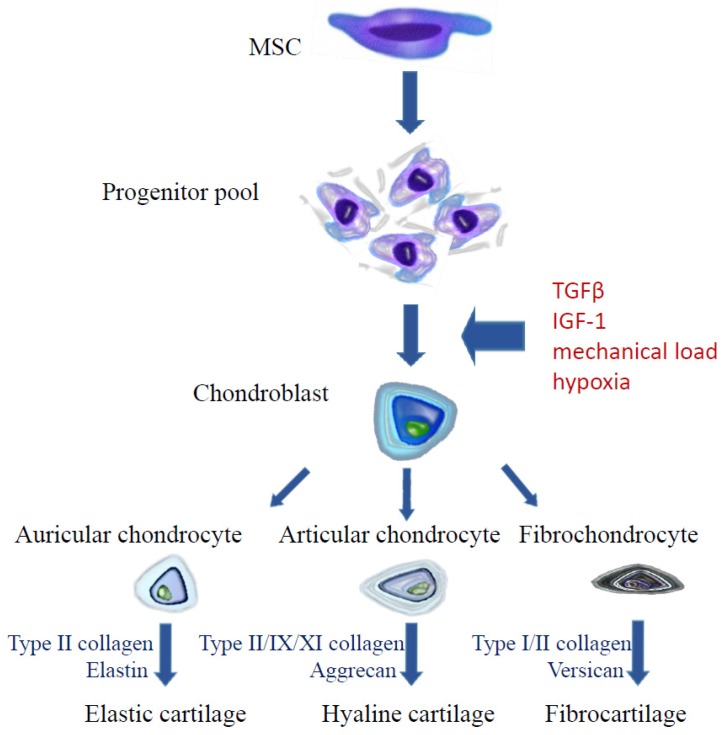
Chondrogenic differentiation of mesenchymal stem cells.

Mesenchymal stem cells are able to differentiate in multiple directions and to self-regenerate [133]. Since MSCs are present in many embryonic tissues (embryonic stem cells, ESC) and in adult individuals (adult stem cells, ASC), there are many methods of acquiring them. Embryonic stem cells can be collected from umbilical cord blood after delivery, from Wharton’s jelly, from the placenta and amniotic fluid and as well as from the subamniotic membrane and perivascular area of the umbilical cord. MSCs have been identified in the following tissues in adult individuals: marrow, adipose tissue, skin, lungs, dental pulp, periosteum, skeletal muscles, tendons and synovial membrane [134] but clinical application of “adult” MSCs is limited mainly to bone marrow-derived mesenchymal stromal cells (BM-MSCs) and adipose-derived stem cells (ADSC, ASC) [135]. According to criteria issued by The International Society for Cellular Therapy (ISCT), characteristic features of mesenchymal cells include the ability to adhere to a plastic base, the presence of three surface antigens: CD105 (endoglin), CD90 (Thy-1), CD73 (ecto-5′-nucleotidase) and concomitant absence of antigens CD45, CD34, CD14 or CD11a, CD79a, or CD19 and class II HLA and the capability of in vitro differentiation towards three cellular lines: osteoblasts, adipocytes and chondroblasts (Figure 2) [133]. Additionally, a detailed description of stem cells includes information, such as the cell origin (tissue, organ, systemic), culture conditions, medium composition, the presence of other antigens of positive identification and absence of negative markers, potential for differentiation, cloning, proteomes, secretomes and transcriptone data [136]. MSCs have a clinically promising immunomodulatory and regenerative potential but it must be noted that MSC also age and die in cultures after several passages [137].

The results of the application of MSCs in regeneration of articular cartilage are promising. In a study by Davatchi et al., intra-articular administration of MSCs to the knee joint (8–9 × 10^6^ cells/patient) resulted in a significant, long term reduction of pain as assessed with the visual analogue scale (VAS), as well as in a reduction of flexion contracture in the joint, of cracking during movements and in an extension of the walking distance [138]. In a randomised, multi-centre clinical trial, a considerable reduction of pain assessed in the VAS and an improvement of the function assessed in the WOMAC (Western Ontario and McMaster Universities OA Index) scale and by measurement of the joint movement range was observed in patients following intra-articular administration of autologous BM-MSCs at 100 × 10^6^ cells/patient in combination with hyaluronic acid. Conversely, an improvement was observed only initially in patients who received BM-MSCs in smaller amounts (10 × 10^6^ cells/patient) but when measured after six months, the change in the parameters under assessment was not significant [139]. An improvement after the administration of even small doses of autologous BM-MSCs into the knee joint (2 × 10^6^ cells/patient) was reported recently by Pers et al. [140]. In the study by Emadedin et al. BM-MSCs at 5 × 10^5^ cells/kg/bw (body weight) were administered to patients with OA of knee joints but also to patients with OA of the ankle and of the hip joint. An improvement of the clinical parameters was observed in these patients for a year but it gradually decreased over the next 18 months [141]. A clinical improvement in all the studies corresponded to that observed in imaging examinations (MRI). Reports published later also confirmed the efficacy and safety of the use of autologous MSCs (Table 1) [138,140,142,143,144,145,146,147,148,149,150,151,152,153,154,155,156]. Use of allogeneic cells could be an alternative to therapy with autologous MSCs (Table 2) [157,158,159,160]. Administration to the knee joint of allogeneic BM-MSCs at 40 × 10^6^ cells/patient [158], 50 × 10^6^ cells/patient, 150 × 10^6^ cells/patient [157] was safe and resulted in a clinical improvement, observed in MRI examinations. Similar results were reported in a recent study by de Windt et al. in which a mixture of allogeneic BM-MSCs and autologous chondrones (chondrocytes with their native pericellular matrix) was used [159,160]. However, a growing body of evidence has been emerging of the time of survival of MSCs at the implantation site being much shorter than expected [161]. Therefore, it cannot be ruled out that MSCs have a regenerative effect on articular cartilage, not only as a source of cells which differentiate towards chondrocytes but also through the secretome that they secrete, which covers a broad spectrum of paracrine factors [162]. After intra-articular administration, MSCs use cytokines to stimulate secretion of extracellular matrix proteases and growth factors, including TGF-β, IGF-1 and FGF [163]. After in vitro addition of articular fluid collected from patients in an early and late stage of OA, larger amounts of factors secreted by MSCs were observed, such as: chemokine (C–X–C motif) ligands, chemokine (C–C motif) ligands and IL-6 in the fluid taken from patients in the initial phase of OA [164]. These findings are indicative of different reactions of MSCs following an intra-articular administration, depending on the degree of local cartilage damage, which can determine the therapeutic indications.

However, there are a number of unknowns associated with the use of MSCs. When administered intra-articularly, MSCs after long culturing may lose their chondrogenic phenotype and die quickly [165]. There have been many attempts at maintaining or restoring the chondrogenic differentiation capability of MSCs, which includes the addition of growth factors, modification of culture conditions and specifying the sources of MSCs; however, there are currently no recommendations in this regard [166,167,168]. Most of the studies conducted to date have been based on MSCs obtained from a patient’s own bone marrow or adipose tissue, which was supposed to limit the immune response; allogeneic MSCs were used only in a few studies. The follow-up period in the published studies conducted to assess the efficacy and safety of intra-articular administration of cultured MSCs (except in studies by Davatchi et al. [138] and Wakitani et al. [145]) was short: from six months to two years. A clinical issue is the tendency of implanted MSCs to differentiate towards fibro-like tissue instead of towards hyaline cartilage [169], which considerably affects the properties of the newly-formed tissue. New sources of MSCs from tissues are sought with a high potential of differentiation towards chondrocytes. Synovial membrane was thought to be such a place. Intra-articular administration of autologous synovium stem cells (SSC) in studies with animal models [170] and in human studies [154,171] proved to cause improvement in imaging and histological parameters of articular cartilage. There are some ongoing studies into maintaining the chondroidal phenotype with advanced methods of genetic modifications and epigenetic regulations.

### 4.3. Cell-Free Procedures

Currently numerous, single-phase scaffold-based cartilage repair techniques exist. Chondral scaffolds used in cartilage repair can be based on components of cartilaginous matrix, such as collagen or hyaluronate [172,173], proteins and natural polymers (such as fibrin, agarose, alginate and chitosan) [174] or synthetic polyesters (such as polylactic acid, polyglycolic acid, polylactide glycolide, polyethylene oxide and polypropylene oxide) [175]. Chondral scaffolds consist of a monolayer material (monophasic scaffold) or they are multi-layer structures, which copy the architecture of cartilage more effectively. Currently, matrices consisting of collagen are used more commonly in regeneration of damaged articular cartilage [118].

As an alternative to cell seeded scaffolds, another treatment approach involves the implantation of acellular biomaterials for cartilage regeneration by stimulating bone marrow stem-cell recruitment and differentiation induced by the scaffold. One of these cell-free procedures is autologous matrix-induced chondrogenesis (AMIC). The method of autologous matrix-induced chondrogenesis is often used in treatment of small, mainly post-traumatic, loss of articular cartilage. AMIC combines stimulation of bone marrow through microfractures with the use of a no-cell membrane made up of type I/III collagen. A collagen membrane stabilises the clot, creates a biological chamber, protects progenitor cells and stimulates the cells to differentiate towards chondrocytes. Collagen membranes are fixed with partial autologous fibrin glue or surgical sutures [176]. Gille et al. showed a significant improvement in all clinical parameters in up to 87% of patients. An analysis of MRI examinations revealed in most cases the total or moderate filling of the cartilage loss with a normal-to-incidentally hyperintense signal. However, an improvement in joint mobility seems to depend on the age of the patients [118]. Moreover, the application of the AMIC technique is recommended in the treatment of local, small cartilage loss and it is contraindicated in cases of multiple cartilage loss, rheumatic diseases and a considerable restriction of joint mobility [176], which considerably limits AMIC applicability in the treatment of aging-related changes.

## 5. Summary

Aging-related changes in articular cartilage lower the possibility of maintaining the properties and regeneration of cartilage. Obviously, time-related factors, such as chondrocytes secretory phenotype, weakening of chondrocytes’ response to growth factors, oxidative stress and accumulation of final glycation products in cartilage can also lead to osteoarthritis, which is frequent in the population of the elderly. Aging of cartilage with time is inevitable and the changes it causes are irreversible. Because of the aging of the global population, age-related diseases of the motor system are a significant social issue. Studies aimed at elucidating the mechanisms of intensified aging of articular cartilage may help to introduce a therapy to slow down the aging-related changes or to support local processes of regeneration of articular cartilage. Due to a growing number of reports published in recent years, increasing attention has been attracted by cell therapies, including the possibility of using MSCs in the regeneration of cartilage. Therapies which employ MSCs offer great potential. However, use of MSCs in the regeneration of aging cartilage raises a lot of doubt. There are no detailed recommendations regarding the culture conditions, the tissue origin and the dosage of stem cells. The degree of changes and the presence of inflammation are the issues which occur in elderly people. Additionally, autologous MSCs in elderly people exhibit lower proliferative activity and there is some difficulty regarding the production of cells with a chondrogenic phenotype and a specific biological activity. However, due to the importance of the issue, further studies are needed of the methods for improving the quality of aging cartilage.

## Figures and Tables

**Table 1:** List of publications of autologous mesenchymal stem cells application for cartilage repair.

Author Year Study Type	Number of Patients	Stem Cell Origin	Number of Cells	Control Group	Follow-up Time	Co-Treatment	Delivery System	Assessments
Wakitani et al. Case and control study [142]	12	Autologous BM-MSCs from iliac crest	1.3 × 10^7^	12 cell free	28–95 weeks	High tibial osteotomy; gel-cell composite; autologous periosteum	Implantation of collagen cell sheets	Arthroscopic photography; Histological samples; Clinical evaluation
Niejadnik et al. Cohort study [143]	36	Autologous BM-MSCs from iliac crest	1.0–1.5 × 10^7^	36 autologous chondrocyte implantation	24 months	Fibrin glue	Implantation upon autologous periosteal patch	ICRS; Lysholm score; Tegner activity scale X-ray; Clinical evaluation
Buda et al. Case series study [144]	20	Autologous BM-MSCs from iliac crest	N/A (2 mL of bone marrow concentrate)	None	24 months	Arthroscopic debridement; Hyaluronic acid membrane scaffold	Transplantation of Arthroscopic Bone-Marrow-Derived Mesenchymal Stem Cells	Clinical evaluation (IKDC, KOOS); MRI; Histochemical analysis
Wakitani et al. Case series [145]	41	Autologous BM-MSCs from iliac crest	5.0 × 10^6^/mL	None	5–137 months	Gel-cell composite	Implantation cell sheets	X-ray; Clinical evaluation
Saw et al. Case series study [146]	5	Autologous peripheral blood progenitor cells	N/A (7–8 mL of peripheral blood progenitor cells	None	10–26 months	Microfracture; Hyaluronic acid intra-articular injections	Intra-articular injection	Second-look arthroscopy; Histological samples;
Davatchi et al. Case series study [147]	4	Autologous BM-MSCs from iliac crest	8.0–9.0 × 10^6^	None	12 months	Saline with 2% human serum albumine	Intra-articular injection	X-ray; Clinical evaluation; VAS
Koh et al. Case and control study [148]	25	Autologous A-MSCs from infrapatellar fat pad	1.2–2.3 × 10^6^	25 cell free	12–18 months	Arthroscopic debridement; Synovectomy	Intra-articular injection	Lysholm score; Tegner activity scale; VAS
Koh et al. Case series study [149]	18	Autologous A-MSCs from infrapatellar fat pad	0.3–2.7 × 10^6^	None	24–26 months	Arthroscopic debridement; Synovectomy; Platelet-rich plasma	Intra-articular injection	Lysholm score; Tegner activity scale; VAS; MRI
Saw et al. Randomised control trial [150]	49	Autologous peripheral blood progenitor cells	N/A (7–8 mL of PBPC)	25 cell free	24 months	Microfracture; Hyaluronic acid intra-articular injections	Intra-articular injection	IKDC; MRI; Second-look arthroscopy; Histological samples
Orozko et al. Case series study [151]	12	Autologous BM-MSCs from iliac crest	40 × 10^6^	None	12 months	Ringer lactate, human albumin, glucose	Intra-articular injection	VAS; Clinical evaluation: WOMAC; MRI
Wong et al. Randomized control trial [152]	28	Autologous BM-MSCs from iliac crest	1.46 × 10^6^	28 cell free	24 months	Microfracture and high tibial osteotomy; Hyaluronic acid;	Intra-articular injection	IKDC; Lysholm score; Tegner activity scale; MOCART
Jo et al. Cohort study [153]	18	Autologous A-MSCs from abdominal subcutaneous fats	1.0 × 10^7^, 5.0 × 10^7^, 1.0 × 10^8^	None	6 months	None	Intra-articular injection	WOMAC; VAS, KSS; X-ray, MRI; Second-look arthroscopy; Histological samples
Akgun et al. Prospective, single-site, randomized, single-blind pilot study. [154]	7	Autologous synovium-derived MSCs	4.0 × 10^6^	7 matrix-induced autologous chondrocyte implantation	24 months	Type I/III collagen membrane (2 cm × 2 cm)	Implantation of MSC preloaded collagen membrane	Clinical evaluation: VAS, KOOS; MRI
Davatchi et al. Case series study [138]	4	Autologous BM-MSCs	8.0–9.0 × 10^6^	None	60 months	Glucosamine was permitted	Intra-articular injection	X-ray; Clinical evaluation: VAS, walking time to pain
Fodor et al. Case series study [155]	6	Adipose-derived stromal vascular cells (the stromal vascular fraction of adipose tissue)	14.0 × 10^6^	None	12 months	None	Intra-articular injection	Clinical evaluation: WOMAC, VAS, ROM, TUG; MRI.
Koh et al. Randomized control trial [156]	40	Autologous A-MSCs	5.0 × 10^6^	40 Microfracture treatment	24 months	Debridement; Microfracture, Fibrin glue	Arthroscopic implantation of MSC loaded in fibrin glue	Second-look arthroscopy; the Lysholm score, KOOS, VAS; MRI; Histological samples
Pers et al. Cohort study [140]	18	Autologous A-MSCs	2.0 × 10^6^, 10.0 × 10^6^, 50.0 × 10^6^	None	6 months	None	Intra-articular injection	WOMAC, VAS, PGA, SAS, KOOS; Histological samples

A-MSCs: adipose-derived mesenchymal stem cells; BM-MSCs: bone marrow-derived mesenchymal stem cells; ICRS: International Cartilage Repair Society score; IKDC: International Knee Documentation Committee; KOOS: the Knee Injury and Osteoarthritis Outcome; KSS: Knee Society Clinical Rating System score; MOCART: Magnetic Resonance Observation of Cartilage Repair Tissue score; MRI: magnetic resonance imaging; MSCs: mesenchymal stem cells; N/A: not available; PGA: Patient Global Assessment; ROM: range of motion; SAS: Short Arthritis Assessment Scale; TUG: timed up-and-go; VAS: visual analogue pain scale; WOMAC: the Western Ontario and McMaster Universities Arthritis Index.

**Table 2:** List of publications of allogenic mesenchymal stem cells application for cartilage repair.

Author Year Study Type	Number of Patients	Stem Cell Origin	Number of Cells	Control Group	Follow-up Time	Co-Treatment	Delivery System	Assessments
Vangsness et al. Randomized, double-blind, controlled study [157]	36	Allogenic BM-MSCs from 18–30-year old donors	5.0 × 10^7^, 1.5 × 10^8^	20 cell free vehicle control	24 months	Partial medial meniscectomy	Intra-articular injection	Measurement of immune cell markers; MRI; VAS; Lysholm score
Vega et al. Randomized control trial [158]	15	Allogenic BM-MSCs	40 × 10^6^	15 Hyaluronic acid (60 mg, single dose)	12 months	None	Intra-articular injection	VAS, WOMAC, SF-12; MRI
De Windt et al. Case series study [159]	10	Allogenic BM-MSCs from the iliac crest of 2 healthy donors (age 2 and 5)	N/A. Mixed cells in the fibrinogen component of fibrin glue at 1.5–2 × 10^6^ cells/mL	10% or 20% autologous chondrons (a standard or a high yield mixture)	12 months	None	Defect site-specific implantation	KOOS, VAS; Second-look arthroscopy; Histological analysis;
De Windt et al. Case series study [160]	35	Allogenic cryopreserved BM-MSCs	N/A. Mixed cells in the fibrinogen component of fibrin glue at 1.5–2 × 10^6^ cells/mL. Approximately 0.9 mL cell product/cm^2^ defect	10% or 20% autologous chondrons (a standard or a high yield mixture)	18 months	None	Defect site-specific implantation	KOOS, VAS; Second-look arthroscopy; Histological analysis;

BM-MSCs: bone marrow-derived mesenchymal stem cells; KOOS: the Knee Injury and Osteoarthritis Outcome; MRI: magnetic resonance imaging; N/A: not available; SF-12: short form-12 life quality questionnaire; VAS: visual analogue pain scale; WOMAC: the Western Ontario and McMaster Universities Arthritis Index.

## References

[1] Bijlsma J.W., Berenbaum F., Lafeber F.P. (2011). Osteoarthritis: An update with relevance for clinical practice. Lancet.

[2] Aigner T., Rose J., Martin J., Buckwalter J. (2004). Aging theories of primary osteoarthritis: From epidemiology to molecular biology. Rejuvenation Res..

[3] Martin J.A., Buckwalter J.A. (2003). The role of chondrocyte senescence in the pathogenesis of osteoarthritis and in limiting cartilage repair. J. Bone Jt. Surg. Am..

[4] Messai H., Duchossoy Y., Khatib A.M., Mitrovic D.R. (2000). Articular chondrocytes from aging rats respond poorly to insulin-like growth factor-1: An altered signalling pathway. Mech. Ageing Dev..

[5] Blanco F.J., Rego I., Ruiz-Romero C. (2011). The role of mitochondria in osteoarthritis. Nat. Rev. Rheumatol..

[6] Huang C.Y., Lai K.Y., Hung L.F., Wu W.L., Liu F.C., Ho L.J. (2011). Advanced glycation end products cause collagen II reduction by activating Janus kinase/signal transducer and activator of transcription 3 pathway in porcine chondrocytes. Rheumatology (Oxford).

[7] Galle J., Bader A., Hepp P., Grill W., Fuchs B., Käs J.A., Krinner A., Marquass B., Müller K., Schiller J. (2010). Mesenchymal Stem Cells in Cartilage Repair: State of the Art and Methods to monitor Cell Growth, Differentiation and Cartilage Regeneration. Curr. Med. Chem..

[8] Hayflick L. (1984). Intracellular determinants of cell aging. Mech. Ageing Dev..

[9] Sikora E., Arendt T., Bennett M., Narita M. (2011). Impact of cellular senescence signature on ageing research. Ageing Res. Rev..

[10] López-Otín C., Blasco M.A., Partridge L., Serrano M., Kroemer G. (2013). The hallmarks of aging. Cell.

[11] Hunt A., Betts D., King W.A., Madan P. (2010). Senescence or apoptosis? The choice bovine fibroblasts make in the presence of increasing concentrations of extracellular H_2_O_2_. SURG J..

[12] Coppe J.P., Desprez P.Y., Krtolica A., Campisi J. (2010). The senescence-associated secretory phenotype: The dark side of tumor suppression. Annu. Rev. Pathol..

[13] Von Zglinicki T., Petrie J., Kirkwood T.B. (2003). Telomere-driven replicative senescence is a stress response. Nat. Biotechnol..

[14] Lu W., Zhang Y., Liu D., Songyang Z., Wan M. (2013). Telomeres-structure, function and regulation. Exp. Cell Res..

[15] Gilley D., Herbert B.S., Huda N., Tanaka H., Reed T. (2008). Factors impacting human telomere homeostasis and age-related disease. Mech. Ageing Dev..

[16] Kaul Z., Cesare A.J., Huschtscha L.I., Neumann A.A., Reddel R.R. (2011). Five dysfunctional telomeres predict onset of senescence in human cells. EMBO Rep..

[17] Bielak-Zmijewska A., Wnuk M., Przybylska D., Grabowska W., Lewinska A., Alster O., Korwek Z., Cmoch A., Myszka A., Pikula S. (2014). A comparison of replicative senescence and doxorubicin-induced premature senescence of vascular smooth muscle cells isolated from human aorta. Biogerontology.

[18] D’Adda di Fagagna F. (2008). Living on a break: Cellular senescence as a DNA-damage response. Nat. Rev. Cancer.

[19] Kühn K., D’Lima D.D., Hashimoto S., Lotz M. (2004). Cell death in cartilage. Osteoarthr. Cartil..

[20] Vignon E., Arlot M., Patricot L.M., Vignon G. (1976). The cell density of human femoral head cartilage. Clin. Orthop. Relat. Res..

[21] Blaney Davidson E.N., Scharstuhl A., Vitters E.L., van der Kraan P.M., van den Berg W.B. (2005). Reduced transforming growth factor-beta signalling in cartilage of old mice: Role in impaired repair capacity. Arthritis Res. Ther..

[22] Aigner T., Hemmel M., Neureiter D., Gebhard P.M., Zeiler G., Kirchner T., McKenna L. (2001). Apoptotic Cell Death Is Not a Widespread Phenomenon in Normal Aging and Osteoarthritis Human Articular Knee Cartilage: A Study of Proliferation, Programmed Cell Death (Apoptosis) and Viability of Chondrocytes in Normal and Osteoarthritic Human Knee Cartilage. Arthritis Rheum..

[23] Ding C., Cicuttini F., Blizzard L., Scott F., Jones G. (2007). A longitudinal study of the effect of sex and age on rate of change in knee cartilage volume in adults. Rheumatology (Oxford).

[24] Wells T., Davidson C., Morgelin M., Bird J.L.E., Bayliss M.T., Dudhia J. (2003). Age-related changes in the composition, the molecular stoichiometry and the stability of proteoglycan aggregates extracted from human articular cartilage. Biochem. J..

[25] Parsch D., Brümmendorf T.H., Richter W., Fellenberg J. (2002). Replicative aging of human articular chondrocytes during ex vivo expansion. Arthritis Rheum..

[26] Harbo M., Delaisse J.M., Kjaersgaard-Andersen P., Soerensen F.B., Koelvraa S., Bendix L. (2013). The relationship between ultra-short telomeres, aging of articular cartilage and the development of human hip osteoarthritis. Mech. Ageing Dev..

[27] Wilson B., Novakofski K.D., Donocoff R.S., Liang Y.X., Fortier L.A. (2014). Telomerase Activity in Articular Chondrocytes Is Lost after Puberty. Cartilage.

[28] Guillot P.V., Gotherstrom C., Chan J., Kurata H., Fisk N.M. (2007). Human first-trimester fetal MSC express pluripotency markers and grow faster and have longer telomeres than adult MSC. Stem Cells.

[29] Mareschi K., Ferrero I., Rustichelli D., Aschero S., Gammaitoni L., Aglietta M., Madon E., Fagioli F. (2006). Expansion of mesenchymal stem cells isolated from pediatric and adult donor bone marrow. J. Cell. Biochem..

[30] Baxter M.A., Wynn R.F., Jowitt S.N., Wraith J.E., Fairbairn L.J., Bellantuono I. (2004). Study of telomere length reveals rapid aging of human marrow stromal cells following in vitro expansion. Stem Cells.

[31] Parsch D., Fellenberg J., Brümmendorf T., Eschlbeck A.M., Richter W. (2004). Telomere length and telomerase activity during expansion and differentiation of human mesenchymal stem cells and chondrocytes. J. Mol. Med..

[32] Dai S.M., Shan Z.Z., Nakamura H., Masuko-Hongo K., Kato T., Nishioka K., Yudoh K. (2006). Catabolic stress induces features of chondrocyte senescence through overexpression of caveolin 1: Possible involvement of caveolin 1-induced down-regulation of articular chondrocytes in the pathogenesis of osteoarthritis. Arthritis Rheum..

[33] Jallali N., Ridha H., Thrasivoulou C., Butler P., Cowen T. (2007). Modulation of intracellular reactive oxygen species level in chondrocytes by IGF-1, FGF and TGF-beta1. Connect. Tissue Res..

[34] Brandl A., Meyer M., Bechmann V., Nerlich M., Angele P. (2011). Oxidative stress induces senescence in human mesenchymal stem cells. Exp. Cell Res..

[35] Harbo M., Bendix L., Bay-Jensen A.C., Graakjaer J., Soe K., Andersen T.L., Kjaersgaard-Andersen P., Koelvraa S., Delaisse J.M. (2012). The distribution pattern of critically short telomeres in human osteoarthritic knees. Arthritis Res. Ther..

[36] Rose J., Söder S., Skhirtladze C., Schmitz N., Gebhard P.M., Sesselmann S., Aigner T. (2012). DNA damage, discoordinated gene expression and cellular senescence in osteoarthritic chondrocytes. Osteoarthr. Cartil..

[37] Brandl A., Hartmann A., Bechmann V., Graf B., Nerlich M., Angele P. (2011). Oxidative stress induces senescence in chondrocytes. J. Orthop. Res..

[38] Loeser R.F. (2011). Aging and osteoarthritis. Curr. Opin. Rheumatol..

[39] Jallali N., Ridha H., Thrasivoulou C., Underwood C., Butler P.E., Cowen T. (2005). Vulnerability to ROS-induced cell death in ageing articular cartilage: The role of antioxidant enzyme activity. Osteoarthr. Cartil..

[40] Yu S.M., Kim S.J. (2013). Thymoquinone-induced reactive oxygen species causes apoptosis of chondrocytes via PI3K/Akt and p38kinase pathway. Exp. Biol. Med..

[41] Yudoh K., van Trieu N., Nakamura H., Hongo-Masuko K., Kato T., Nishioka K. (2005). Potential involvement of oxidative stress in cartilage senescence and development of osteoarthritis: Oxidative stress induces chondrocyte telomere instability and downregulation of chondrocyte function. Arthritis Res. Ther..

[42] Ashraf S., Cha B.H., Kim J.S., Ahn J., Han I., Park H., Lee S.H. (2016). Regulation of senescence associated signalling mechanisms in chondrocytes for cartilage tissue regeneration. Osteoarthr. Cartil..

[43] Loeser R.F., Gandhi U., Long D.L., Yin W., Chubinskaya S. (2014). Aging and oxidative stress reduce the response of human articular chondrocytes to insulin-like growth factor 1 and osteogenic protein 1. Arthritis Rheumatol..

[44] Li C.J., Sun L.Y., Pang C.Y. (2015). Synergistic Protection of N-acetylcysteine and ascorbic acid 2-phosphate on human mesenchymal stem cells against mitoptosis, necroptosis and apoptosis. Sci. Rep..

[45] Sakata S., Hayashi S., Fujishiro T., Kawakita K., Kanzaki N., Hashimoto S., Iwasa K., Chinzei N., Kihara S., Haneda M. (2015). Oxidative stress-induced apoptosis and matrix loss of chondrocytes is inhibited by eicosapentaenoic acid. J. Orthop. Res..

[46] Lin T.M., Tsai J.L., Lin S.D., Lai C.S., Chang C.C. (2005). Accelerated growth and prolonged lifespan of adipose tissue-derived human mesenchymal stem cells in a medium using reduced calcium and antioxidants. Stem Cells Dev..

[47] Estrada J.C., Torres Y., Benguria A., Dopazo A., Roche E., Carrera-Quintanar L., Pérez R.A., Enríquez J.A., Torres R., Ramírez J.C. (2013). Human mesenchymal stem cell-replicative senescence and oxidative stress are closely linked to aneuploidy. Cell Death Dis..

[48] Munir S., Foldager C., Lind M., Zachar V., Søballe K., Koch T. (2014). Hypoxia enhances chondrogenic differentiation of human adipose tissue-derived stromal cells in scaffold-free and scaffold systems. Cell. Tissue Res..

[49] Lomri A. (2008). Role of reactive oxygen species and superoxide dismutase in cartilage aging and pathology. Future Reumatol..

[50] Greene M.A., Loeser R.F. (2015). Aging-related inflammation in osteoarthritis. Osteoarthr. Cartil..

[51] Livshits G., Zhai G., Hart D.J., Kato B.S., Wang H., Williams F.M., Spector T.D. (2009). Interleukin-6 is a significant predictor of radiographic knee osteoarthritis: The Chingford study. Arthritis Rheum..

[52] Philipot D., Guerit D., Platano D., Chuchana P., Olivotto E., Espinoza F., Dorandeu A., Pers Y.M., Piette J., Borziet R.M. (2014). p16INK4a and its regulator miR-24 link senescence and chondrocyte terminal differentiation-associated matrix remodeling in osteoarthritis. Arthritis Res. Ther..

[53] Wu W., Billinghurst R.C., Pidoux I., Antoniou J., Zukor D., Tanzer M., Poole A.R. (2002). Sites of collagenase cleavage and denaturation of type II collagen in aging and osteoarthritic articular cartilage and their relationship to the distribution of matrix metalloproteinase 1 and matrix metalloproteinase 13. Arthritis Rheum..

[54] Aurich M., Poole A.R., Reiner A., Mollenhauer C., Margulis A., Kuettner K.E., Cole A.A. (2002). Matrix homeostasis in aging normal human ankle cartilage. Arthritis Rheum..

[55] Forsyth C.B., Cole A., Murphy G., Bienias J.L., Im H.J., Loeser R.F. (2005). Increased matrix metalloproteinase-13 production with aging by human articular chondrocytes in response to catabolic stimuli. J. Gerontol. A Biol. Sci. Med. Sci..

[56] Long D., Blake S., Song X.Y., Lark M., Loeser R.F. (2008). Human articular chondrocytes produce IL-7 and respond to IL-7 with increased production of matrix metalloproteinase-13. Arthritis Res. Ther..

[57] Loeser R.F. (2009). Aging and osteoarthritis: The role of chondrocyte senescence and aging changes in the cartilage matrix. Osteoarthr. Cartil..

[58] Hashimoto M., Nakasa T., Hikata T., Asahara H. (2008). Molecular network of cartilage homeostasis and osteoarthritis. Med. Res. Rev..

[59] Goldberg A. (2001). Effects of growth factors on articular cartilage. Ortop. Traumatol. Rehab..

[60] De Ceuninck F., Caliez A., Dassencourt L., Anract P., Renard P. (2004). Pharmacological disruption of insulin-like growth factor 1 binding to IGF-binding proteins restores anabolic responses in human osteoarthritic chondrocytes. Arthritis Res. Ther..

[61] Yin W., Park J.I., Loeser R.F. (2009). Oxidative stress inhibits insulin-like growth factor-I induction of chondrocyte proteoglycan synthesis through differential regulation of phosphatidylinositol 3-Kinase-Akt and MEK-ERK MAPK signaling pathways. J. Biol. Chem..

[62] Starkman B.G., Cravero J.D., Delcarlo M., Loeser R.F. (2005). IGF-I stimulation of proteoglycan synthesis by chondrocytes requires activation of the PI 3-kinase pathway but not ERK MAPK. Biochem. J..

[63] Allan E.H., Martin T.J. (1995). The plasminogen activator inhibitor system in bone cell function. Clin. Orthop. Relat. Res..

[64] Murphy-Ullrich J.E., Poczatek M. (2000). Activation of latent TGF-beta by thrombospondin-1: Mechanisms and physiology. Cytokine Growth Factor Rev..

[65] Dangelo M., Sarment D.P., Billings P.C., Pacifici M. (2001). Activation of transforming growth factor beta in chondrocytes undergoing endochondral ossification. J. Bone Miner. Res..

[66] Albro M.B., Cigan A.D., Nims R.J., Yeroushalmi K.J., Oungoulian S.R., Hung C.T., Ateshian G.A. (2012). Shearing of synovial fluid activates latent TGF-β. Osteoarthr. Cartil..

[67] Gordon K.J., Blobe G.C. (2008). Role of transforming growth factor-beta superfamily signaling pathways in human disease. Biochim. Biophys. Acta.

[68] Blaney Davidson E.N., Remst D.F., Vitters E.L., van Beuningen H.M., Blom A.B., Goumans M.J., van den Berg W.B., van der Kraan P.M. (2009). Increase in ALK1/ALK5 ratio as a cause for elevated MMP-13 expression in osteoarthritis in humans and mice. J. Immunol..

[69] Finnson K.W., Parker W.L., ten Dijke P., Thorikay M., Philip A. (2008). ALK1 opposes ALK5/Smad3 signaling and expression of extracellular matrix components in human chondrocytes. J. Bone Miner. Res..

[70] Li W., Ding S. (2010). Generation of novel rat and human pluripotent stem cells by reprogramming and chemical approaches. Methods Mol. Biol..

[71] Johnstone B., Hering T.M., Caplan A.I., Goldberg V.M., Yoo J.U. (1998). In vitro chondrogenesis of bone marrow-derived mesenchymal progenitor cells. Exp. Cell Res..

[72] Wu J., Niu J., Li X., Wang X., Guo Z., Zhang F. (2014). TGF-β1 induces senescence of bone marrow mesenchymal stem cells via increase of mitochondrial ROS production. BMC Dev. Biol..

[73] Ahmed U., Anwar A., Savage R.S., Thornalley P.J., Rabbani N. (2016). Protein oxidation, nitration and glycation biomarkers for early-stage diagnosis of osteoarthritis of the knee and typing and progression of arthritic disease. Arthritis Res. Ther..

[74] Saudek D.M., Kay J. (2003). Advanced glycation endproducts and osteoarthritis. Curr. Reumatol. Rep..

[75] Mizumura K., Choi A.M., Ryster S.W. (2014). Emerging role of selective autophagy in human diseases. Front. Pharmacol..

[76] Srinivas V., Bohensky J., Shapiro I.M. (2009). Autophagy: A new phase in the maturation of growth plate chondrocytes is regulated by HIF, mTOR and AMP kinase. Cells Tissues Organs.

[77] Shapiro I.M., Layfield R., Lotz M., Settembre C., Whitehouse C. (2014). Boning up on autophagy: The role of autophagy in skeletal biology. Autophagy.

[78] Carames B., Taniguchi N., Otsuki S., Blanco F.J., Lotz M. (2010). Autophagy is a protective mechanism in normal cartilage and its aging-related loss is linked with cell death and osteoarthritis. Arthritis Rheum..

[79] Zhang Y., Vasheghani F., Li Y.H., Blati M., Simeone K., Fahmi H., Lussier B., Roughley P., Lagares D., Pelletier J.P. (2015). Cartilage-specific deletion of mTOR upregulates autophagy and protects mice from osteoarthritis. Ann. Rheum. Dis..

[80] Takayama K., Kawakami Y., Kobayashi M., Greco N., Cummins J.H., Matsushita T., Kuroda R., Kurosaka M., Fu F.H., Huard J. (2014). Local intra-articular injection of rapamycin delays articular cartilage degeneration in a murine model of osteoarthritis. Arthritis Res. Ther..

[81] Matsuzaki T., Matsushita T., Tabata Y., Saito T., Matsumoto T., Nagai K., Kuroda R., Kurosaka M. (2014). Intra-articular administration of gelatin hydrogels incorporating rapamycin-micelles reduces the development of experimental osteoarthritis in a murine model. Biomaterials.

[82] Alvarez-Garcia O., Olmer M., Akagi R., Akasaki Y., Fisch K.M., Shen T., Su A.I., Lotz M.K. (2016). Suppression of REDD1 in osteoarthritis cartilage, a novel mechanism for dysregulated mTOR signaling and defective autophagy. Osteoarthr. Cartil..

[83] Zhang F.J., Luo W., Lei G.H. (2015). Role of HIF-1alpha and HIF-2alpha in osteoarthritis. Jt. Bone Spine.

[84] Bohensky J., Terkhorn S.P., Freeman T.A., Adams C.S., Garcia J., Shapiro I.M., Srinivas V. (2009). Regulation of autophagy in humanand murine cartilage: Hypoxia-inducible factor 2 suppresses chondrocyteautophagy. Arthritis Rheum..

[85] Simonsen J.L., Rosada C., Serakinci N., Justesen J., Stenderup K., Rattan S.I., Jensen T.G., Kassem M. (2002). Telomerase expression extends the proliferative life-span and maintains the osteogenic potential of human bone marrow stromal cells. Nat. Biotechnol..

[86] Shi S., Gronthos S., Chen S., Reddi A., Counter C.M., Robey P.G., Wang C.Y. (2002). Bone formation by human postnatal bone marrow stromal stem cells is enhanced by telomerase expression. Nat. Biotechnol..

[87] Pearce V.P., Sherrell J., Lou Z., Kopelovich L., Wright W.E., Shay J.W. (2008). Immortalization of epithelial progenitor cells mediated by resveratrol. Oncogene.

[88] Sprouse A.A., Steding C.E., Herbert B.S. (2012). Pharmaceutical regulation of telomerase and its clinical potential. J. Cell. Mol. Med..

[89] Shen C.Y., Jiang J.G., Yang L., Wang D.W., Zhu W. (2017). Anti-aging active ingredients from herbs and nutraceuticals used in TCM: Pharmacological mechanisms and implications for drug discovery. Br. J. Pharmacol..

[90] Yung L.Y., Lam W.S., Ho M.K., Hu Y., Ip F.C., Pang H., Chin A.C., Harley C.B., Ip N.Y., Wong Y.H. (2012). Astragaloside IV and cycloastragenol stimulate the phosphorylation of extracellular signal-regulated protein kinase in multiple cell types. Planta Med..

[91] Meng H.C., Wang S., Li Y., Kuang Y.Y., Ma C.M. (2013). Chemical constituents and pharmacologic actions of Cynomorium plants. Chin. J. Nat. Med..

[92] Tichon A., Eitan E., Kurkalli B.G., Braiman A., Gazit A., Slavin S., Beith-Yannai E., Priel E. (2013). Oxidative stress protection by novel telomerase activators in mesenchymal stem cells derived from healthy and diseased individuals. Curr. Mol. Med..

[93] Taka T., Changtam C., Thaichana P., Kaewtunjai N., Suksamrarn A., Lee T.R., Tuntiwechapikul W. (2014). Curcuminoid derivatives enhance telomerase activity in an in vitro TRAP assay. Bioorg. Med. Chem. Lett..

[94] Molgora B., Bateman R., Sweeney G., Finger D., Dimler T., Effros R.B., Valenzuela H.F. (2013). Functional assessment of pharmacological telomerase activators in human T cells. Cells.

[95] De Jesus B.B., Schneeberger K., Vera E., Tejera A., Harley C.B., Blasco M.A. (2011). The telomerase activator TA-65 elongates short telomeres and increases health span of adult/old mice without increasing cancer incidence. Aging Cell..

[96] Liu L., Gu H., Liu H., Jiao Y., Li K., Zhao Y., An L., Yang J. (2014). Protective effect of resveratrol against IL-1β-induced inflammatory response on human osteoarthritic chondrocytes partly via the TLR4/MyD88/NF-κB signaling pathway: An “in vitro study”. Int. J. Mol. Sci..

[97] Li W., Cai L., Zhang Y., Cui L., Shen G. (2015). Intra-articular resveratrol injection prevents osteoarthritis progression in a mouse model by activating SIRT1 and thereby silencing HIF-2α. J. Orthop. Res..

[98] Toh W.S., Loh X.J. (2014). Advances in hydrogel delivery systems for tissue regeneration. Mater. Sci. Eng. C. Mater. Biol. Appl..

[99] Wang W., Sun L., Zhang P., Song J., Liu W. (2014). An anti-inflammatory cell-free collagen/resveratrol scaffold for repairing osteochondral defects in rabbits. Acta Biomater..

[100] Moussavi-Harami F., Duwayri Y., Martin J.A., Moussavi-Harami F., Buckwalter J.A. (2004). Oxygen effects on senescence in chondrocytes and mesenchymal stem cells: Consequences for tissue engineering. Iowa Orthop. J..

[101] Martin J.A., Klingelhutz A.J., Moussavi-Harami F., Buckwalter J.A. (2004). Effects of oxidative damage and telomerase activity on human articular cartilage chondrocyte senescence. J. Gerontol. A. Biol. Sci. Med. Sci..

[102] Egli R.J., Bastian J.D., Ganz R., Hofstetter W., Leunig M. (2008). Hypoxic expansion promotes the chondrogenic potential of articular chondrocytes. J. Orthop. Res..

[103] Schrobback K., Klein T.J., Crawford R., Upton Z., Malda J., Leavesley D.I. (2012). Effects of oxygen and culture system on in vitro propagation and redifferentiation of osteoarthritic human articular chondrocytes. Cell Tissue Res..

[104] Choi J.R., Pingguan-Murphy B., Wan Abas W.A.B., Noor Azmi M.A., Omar S.Z., Chua K.H., Wan Safwani W.K. (2014). Impact of low oxygen tension on stemness, proliferation and differentiation potential of human adipose-derived stem cells. Biochem. Biophys. Res. Commun..

[105] Tsai C.C., Chen Y.J., Yew T.L., Chen L.L., Wang J.Y., Chiu C.H., Hung S.C. (2011). Hypoxia inhibits senescence and maintains mesenchymal stem cell properties through down-regulation of E2A-p21 by HIF-TWIST. Blood.

[106] Wan Safwani W.K.Z., Choi J.R., Yong K.W., Ting I., Mat Adenan N.A., Pingguan-Murphy B. (2017). Hypoxia enhances the viability, growth and chondrogenic potential of cryopreserved human adipose-derived stem cells. Cryobiology.

[107] Wan Safwani W.K.Z., Wong C.W., Yong K.W., Choi J.R., Mat Adenan N.A., Omar S.Z., Wan Abas W.A.B., Pingguan-Murphy B. (2013). The effects of hypoxia and serum-free conditions on the stemness properties of human adipose-derived stem cells. Cytotechnology.

[108] Öztürk E., Hobiger S., Despot-Slade E., Pichler M., Zenobi-Wong M. (2017). Hypoxia regulates RhoA and Wnt/β-catenin signaling in a context-dependent way to control re-differentiation of chondrocytes. Sci. Rep..

[109] Grodzinsky A.J., Levenston M.E., Jin M., Frank E.H. (2000). Cartilage tissue remodeling in response to mechanical forces. Annu. Rev. Biomed Eng..

[110] Schätti O., Grad S., Goldhahn J., Salzmann G., Li Z., Alini M., Stoddart M.J. (2011). A combination of shear and dynamic compression leads to mechanically induced chondrogenesis of human mesenchymal stem cells. Eur. Cell Mater..

[111] Gardner O.F., Fahy N., Alini M., Stoddart M.J. (2016). Differences in human mesenchymal stell cell secretomes during chondrogenic induction. Eur. Cell Mater..

[112] Li Z., Kupcsik L., Yao S.J., Alini M., Stoddart M.J. (2010). Mechanical Load Modulates Chondrogenesis of Human Mesenchymal Stem Cells through the TGFβ Pathway. J. Cell Mol. Med..

[113] Guo T., Yu L., Lim C.G., Goodley A.S., Xiao X., Placone J.K., Ferlin K.M., Nguyen B.N., Hsieh A.H., Fisher J.P. (2016). Effect of Dynamic Culture and Periodic Compression on Human Mesenchymal Stem Cell Proliferation and Chondrogenesis. Ann. Biomed. Eng..

[114] Bian L., Zhai D.Y., Zhang E.C., Mauck R.L., Burdick J.A. (2012). Dynamic compressive loading enhances cartilage matrix synthesis and distribution and suppresses hypertrophy in hMSC-laden hyaluronic acid hydrogels. Tissue Eng. Part A.

[115] Zhang T., Wen F., Wu Y., Goh G.S., Ge Z., Tan L.P., Hui J.H., Yang Z. (2015). Cross-talk between TGF-beta/SMAD and integrin signaling pathways in regulating hypertrophy of mesenchymal stem cell chondrogenesis under deferral dynamic compression. Biomaterials.

[116] Simon T.M., Jackson D.W. (2018). Articular Cartilage: Injury Pathways and Treatment Options. Sports Med. Arthrosc. Rev..

[117] Al-Najar M., Khalil H., Al-Ajlouni J., Al-Antary E., Hamdan M., Rahmeh R., Alhattab D., Samara O., Yasin M., Abdullah A.A. (2017). Intra-articular injection of expanded autologous bone marrow mesenchymal cells in moderate and severe knee osteoarthritis is safe: A phase I/II study. J. Orthop. Surg. Res..

[118] Gille J., Behrens P., Volpi P., de Girolamo L., Reiss E., Zoch W., Anders S. (2013). Outcome of Autologous Matrix Induced Chondrogenesis (AMIC) in cartilage knee surgery: Data of the AMIC Registry. Arch. Orthop. Trauma Surg..

[119] Jo C.H., Chai J.W., Jeong E.C., Oh S., Shin J.S., Shim H., Yoon K.S. (2017). Intra-articular Injection of Mesenchymal Stem Cells for the Treatment of Osteoarthritis of the Knee: A 2-Year Follow-up Study. Am. J. Sports Med..

[120] Brittberg M., Lindahl A., Nilsson A., Ohlsson C., Isaksson O., Peterson L. (1994). Treatment of deep cartilage defects in the knee with autologous chondrocyte transplantation. N. Engl. J. Med..

[121] Peterson L., Brittberg M., Kiviranta I., Akerlund E.L., Lindahl A. (2002). Autologous chon-drocyte transplantation. Biomechanics and long-term durability. Am. J. Sports Med..

[122] Li Y., Wei X., Zhou J., Wei L. (2013). The age-related changes in cartilage and osteoarthritis. BioMed Res. Int..

[123] Barbero A., Grogan S., Schafer D., Heberer M., Mainil-Varlet P., Martin I. (2004). Age related changes in human articular chondrocyte yield, proliferation and post-expansion chondrogenic capacity. Osteoarthr. Cartil..

[124] Schulze-Tanzil G. (2009). Activation and dedifferentiation of chondrocytes: Implications in cartilage injury and repair. Ann. Anat..

[125] Caron M.M., Emans P.J., Coolsen M.M., Voss L., Surtel D.A., Cremers A., van Rhijn L.W., Welting T.J. (2012). Redifferentiation of dedifferentiated human articular chondrocytes: Comparison of 2D and 3D cultures. Osteoarthr. Cartil..

[126] Meinert C., Schrobback K., Hutmacher D.W., Klein T.J. (2017). A novel bioreactor system for biaxial mechanical loading enhances the properties of tissue-engineered human cartilage. Sci. Rep..

[127] Rosenzweig D.H., Solar-Cafaggi S., Quinn T.M. (2012). Functionalization of dynamic culture surfaces with a cartilage extracellular matrix extract enhances chondrocyte phenotype against dedifferentiation. Acta Biomater..

[128] Rosenzweig D.H., Matmati M., Khayat G., Chaudhry S., Hinz B., Quinn T.M. (2012). Culture of primary bovine chondrocytes on a continuously expanding surface inhibits dedifferentiation. Tissue Eng. Part A.

[129] Kino-Oka M., Yashiki S., Ota Y., Mushiaki Y., Sugawara K., Yamamoto T., Takezawa T., Taya M. (2005). Subculture of chondrocytes on a collagen type I-coated substrate with suppressed cellular dedifferentiation. Tissue Eng..

[130] Das R.H., Jahr H., Verhaar J.A., van der Linden J.C., van Osch G.J., Weinans H. (2008). In vitro expansion affects the response of chondrocytes to mechanical stimulation. Osteoarthr. Cartil..

[131] Barbero A., Martin I. (2007). Human articular chondrocytes culture. Methods Mol. Med..

[132] Gurusinghe S., Strappe P. (2014). Gene modification of mesenchymal stem cells and articular chondrocytes to enhance chondrogenesis. BioMed Res. Int..

[133] Dominici M., Le Blanc K., Mueller I., Slaper-Cortenbach I., Marini F., Krause D., Deans R., Keating A., Prockop D.J., Horwitz E. (2006). Minimal criteria for defining multipotent mesenchymal stromal cells. The International Society for Cellular Therapy position statement. Cytotherapy.

[134] Girlovanu M., Susman S., Soritau O., Rus-Ciuca D., Melincovici C., Constantin A.M., Mihu C.M. (2015). Stem cells—Biological update and cell therapy progress. Clujul. Med..

[135] Im G.I. (2017). Bone marrow-derived stem/stromal cells and adipose tissue-derived stem/stromal cells: Their comparative efficacies and synergistic effects. J. Biomed. Mater. Res. A.

[136] Keating A. (2012). Mesenchymal stromal cells: New directions. Cell Stem Cell.

[137] Ho A.D., Wagner W., Franke W. (2008). Heterogeneity of mesenchymal stromal cell preparations. Cytotherapy.

[138] Davatchi F., Sadeghi Abdollahi B., Mohyeddin M., Nikbin B. (2016). Mesenchymal stem cell therapy for knee osteoarthritis: 5 years follow-up of three patients. Int. J. Rheum. Dis..

[139] Lamo-Espinosa J.M., Mora G., Blanco J.F., Granero-Moltó F., Nuñez-Córdoba J.M., Sánchez-Echenique C., Bondía J.M., Aquerreta J.D., Andreu E.J., Ornilla E. (2016). Intra-articular injection of two different doses of autologous bone marrow mesenchymal stem cells versus hyaluronic acid in the treatment of knee osteoarthritis: Multicenter randomized controlled clinical trial (phase I/II). J. Transl. Med..

[140] Pers Y.M., Rackwitz L., Ferreira R., Pullig O., Delfour C., Barry F., Sensebe L., Casteilla L., Fleury S., Bourin P. (2016). Adipose Mesenchymal Stromal Cell-Based Therapy for Severe Osteoarthritis of the Knee: A Phase I Dose-Escalation Trial. Stem Cells Transl. Med..

[141] Emadedin M., Ghorbani Liastani M., Fazeli R., Mohseni F., Moghadasali R., Mardpour S., Hosseini S.E., Niknejadi M., Moeininia F., Aghahossein Fanni A. (2015). Long-Term Follow-up of Intra-articular Injection of Autologous Mesenchymal Stem Cells in Patients with Knee, Ankle, or Hip Osteoarthritis. Arch. Iran. Med..

[142] Wakitani S., Imoto K., Yamamoto T., Saito M., Murata N., Yoneda M. (2002). Human autologous culture expanded bone marrow mesenchymal cell transplantation for repair of cartilage defects in osteoarthritic knees. Osteoarthr. Cartil..

[143] Nejadnik H., Hui J.H., Feng Choong E.P., Tai B.C., Lee E.H. (2010). Autologous bone marrow-derived mesenchymal stem cells versus autologous chondrocyte implantation: An observational cohort study. Am. J. Sports Med..

[144] Buda R., Vannini F., Cavallo M., Grigolo B., Cenacchi A., Giannini S. (2010). Osteochondral lesions of the knee: A new one-step repair technique with bone-marrow-derived cells. J. Bone Jt. Surg. Am..

[145] Wakitani S., Okabe T., Horibe S., Mitsuoka T., Saito M., Koyama T., Nawata M., Tensho K., Kato H., Uematsu K. (2011). Safety of autologous bone marrow-derived mesenchymal stem cell transplantation for cartilage repair in 41 patients with 45 joints followed for up to 11 years and 5 months. J. Tissue Eng. Regen. Med..

[146] Saw K.Y., Anz A., Merican S., Tay Y.G., Ragavanaidu K., Jee C.S., McGuire D.A. (2011). Articular cartilage regeneration with autologous peripheral blood progenitor cells and hyaluronic acid after arthroscopic subchondral drilling: A report of 5 cases with histology. Arthroscopy.

[147] Davatchi F., Abdollahi B.S., Mohyeddin M., Shahram F., Nikbin B. (2011). Mesenchymal stem cell therapy for knee osteoarthritis. Preliminary report of four patients. Int. J. Rheum. Dis..

[148] Koh Y.G., Choi Y.J. (2012). Infrapatellar fat pad-derived mesenchymal stem cell therapy for knee osteoarthritis. Knee.

[149] Koh Y.G., Jo S.B., Kwon O.R., Suh D.S., Lee S.W., Park S.H., Choi Y.J. (2013). Mesenchymal stem cell injections improve symptoms of knee osteoarthritis. Arthroscopy.

[150] Saw K.Y., Anz A., Siew-Yoke Jee C., Merican S., Ching-Soong Ng R., Roohi S.A., Ragavanaidu K. (2013). Articular cartilage regeneration with autologous peripheral blood stem cells versus hyaluronic acid: A randomized controlled trial. Arthroscopy.

[151] Orozco L., Munar A., Soler R., Alberca M., Soler F., Huguet M., Sentís J., Sánchez A., García-Sancho J. (2013). Treatment of knee osteoarthritis with autologous mesenchymal stem cells: A pilot study. Transplantation.

[152] Wong K.L., Lee K.B., Tai B.C., Law P., Lee E.H., Hui J.H. (2013). Injectable cultured bone marrow-derived mesenchymal stem cells in varus knees with cartilage defects undergoing high tibial osteotomy: A prospective, randomized controlled clinical trial with 2 years’ follow-up. Arthroscopy.

[153] Jo C.H., Lee Y.G., Shin W.H., Kim H., Chai J.W., Jeong E.C., Kim J.E., Shim H., Shin J.S., Shin I.S. (2014). Intra-articular injection of mesenchymal stem cells for the treatment of osteoarthritis of the knee: A proof-of-concept clinical trial. Stem Cells.

[154] Akgun I., Unlu M.C., Erdal O.A., Ogut T., Erturk M., Ovali E., Kantarci F., Caliskan G., Akgun Y. (2015). Matrix-induced autologous mesenchymal stem cell implantation versus matrix-induced autologous chondrocyte implantation in the treatment of chondral defects of the knee: A 2-year randomized study. Arch. Orthop. Trauma Surg..

[155] Fodor P.B., Paulseth S.G. (2016). Adipose derived stromal cell (ADSC) injections for pain management of osteoarthritis in the human knee joint. Aesthet. Surg. J..

[156] Koh Y.G., Kwon O.R., Kim Y.S., Choi Y.J., Tak D.H. (2016). Adipose-Derived mesenchymal stem cells with microfracture versus microfracture alone: 2-year follow-up of a prospective randomized trial. Arthroscopy.

[157] Vangsness C.T., Farr J., Boyd J., Dellaero D.T., Mills C.R., LeRoux-Williams M. (2014). Adult human mesenchymal stem cells delivered via intra-articular injection to the knee following partial medial meniscectomy: A randomized, double-blind, controlled study. J. Bone Jt. Surg. Am..

[158] Vega A., Martin-Ferrero M.A., del Canto F., Alberca M., Garcia V., Munar A., Orozco L., Soler R., Fuertes J.J., Huguet M. (2015). Treatment of knee osteoarthritis with allogeneic bone marrow mesenchymal stem cells: A randomized controlled trial. Transplantation.

[159] De Windt T.S., Vonk L.A., Slaper-Cortenbach I.C., van den Broek M.P., Nizak R., van Rijen M.H., de Weger R.A., Dhert W.J., Saris D.B. (2017). Allogeneic Mesenchymal Stem Cells Stimulate Cartilage Regeneration and Are Safe for Single-Stage Cartilage Repair in Humans upon Mixture with Recycled Autologous Chondrons. Stem Cells.

[160] De Windt T.S., Vonk L.A., Slaper-Cortenbach I.C.M., Nizak R., van Rijen M.H.P., Saris D.B.F. (2017). Allogeneic MSCs and Recycled Autologous Chondrons Mixed in a One-Stage Cartilage Cell Transplantion: A First-in-Man Trial in 35 Patients. Stem Cells.

[161] Baldari S., Di Rocco G., Piccoli M., Pozzobon M., Muraca M., Toietta G. (2017). Challenges and Strategies for Improving the Regenerative Effects of Mesenchymal Stromal Cell-Based Therapies. Int. J. Mol. Sci..

[162] Liang X., Ding Y., Zhang Y., Tse H.F., Lian Q. (2014). Paracrine mechanisms of mesenchymal stem cell-based therapy: Current status and perspectives. Cell Transpl..

[163] Steinert A.F., Rackwitz L., Gilbert F., Noth U., Tuan R.S. (2012). Concise review: The clinical application of mesenchymal stem cells for musculoskeletal regeneration: Current status and perspectives. Stem Cells Transl. Med..

[164] Gomez-Aristizabal A., Sharma A., Bakooshli M.A., Kapoor M., Gilbert P.M., Viswanathan S., Gandhi R. (2017). Stage-specific differences in secretory profile of mesenchymal stromal cells (MSCs) subjected to early- vs. late-stage OA synovial fluid. Osteoarthr. Cartil..

[165] Farrell M.J., Fisher M.B., Huang A.H., Shin J.I., Farrell K.M., Mauck R.L. (2014). Functional properties of bone marrow-derived MSC-based engineered cartilage are unstable with very longterm in vitro culture. J. Biomech..

[166] Perez-Silos V., Camacho-Morales A., Fuentes-Mera L. (2016). Mesenchymal stem cells subpopulations: Application for orthopedic regenerative medicine. Stem Cells Int..

[167] Clause K.C., Liu L.J., Tobita K. (2010). Directed stem cell differentiation: The role of physical forces. Cell Commun. Adhes..

[168] Gugjoo M.B., Amarpal, Sharma G.T., Aithal H.P., Kinjavdekar P. (2016). Cartilage tissue engineering: Role of mesenchymal stem cells along with growth factors & scaffolds. Indian J. Med. Res..

[169] Vinardell T., Sheehy E.J., Buckley C.T., Kelly D.J. (2012). A comparison of the functionality and in vivo phenotypic stability of cartilaginous tissues engineered from different stem cell sources. Tissue Eng. Part A.

[170] Mak J., Jablonski C.L., Leonard C.A., Dunn J.F., Raharjo E., Matyas J.R., Biernaskie J., Krawetz R.J. (2016). Intra-articular injection of synovial mesenchymal stem cells improves cartilage repair in a mouse injury model. Sci. Rep..

[171] Sekiya I., Muneta T., Horie M., Koga H. (2015). Arthroscopic Transplantation of Synovial Stem Cells Improves Clinical Outcomes in Knees with Cartilage Defects. Clin. Orthop. Relat. Res..

[172] Van Osch G.J., Brittberg M., Dennis J.E., Bastiaansen-Jenniskens Y.M., Erben R.G., Konttinen Y.T., Luyten F.P. (2009). Cartilage repair: Past and future—Lessons for regenerative medicine. J. Cell. Mol. Med..

[173] Gomoll A.H. (2012). Microfracture and augments. J. Knee Surg..

[174] Bonzani I.C., George J.H., Stevens M.M. (2006). Novel materials for bone and cartilage regeneration. Curr. Opin. Chem. Biol..

[175] Kon E., Roffi A., Filardo G., Tesei G., Marcacci M. (2015). Scaffold-based cartilage treatments: With or without cells? A systematic review of preclinical and clinical evidence. Arthroscopy.

[176] Benthien J.P., Behrens P. (2010). Autologous Matrix-Induced Chondrogenesis (AMIC): Combining Microfracturing and a Collagen I/III Matrix for Articular Cartilage Resurfacing. Cartilage.

